# Azide-functionalized SpCas9 enables generation of site-selective and bioactive Cas9-siRNA conjugates

**DOI:** 10.1039/d6cc01443g

**Published:** 2026-06-02

**Authors:** Danny Wilbie, Matt Timmers, Asimina Kogkalidou, Erik R. Hebels, Igor R. Sweet, Roel Maas-Bakker, Esmeralda Bosman, Olivier G. de Jong, Tina Vermonden, Enrico Mastrobattista

**Affiliations:** a Division of Pharmaceutics, Utrecht Institute for Pharmaceutical Sciences (UIPS), Utrecht University Utrecht 3508 TB The Netherlands e.mastrobattista@uu.nl; b Cristal Therapeutics Maastricht 6229 EV The Netherlands; c Division of Chemical Biology and Drug Discovery, Utrecht Institute for Pharmaceutical Sciences, Utrecht University Utrecht 3508 TB The Netherlands

## Abstract

The gene editing enzyme SpCas9 was engineered to display azide on its surface, enabling azide–alkyne click conjugation. As a proof of concept, siRNA functionalized with a reduction sensitive linker and ring-strained alkyne tetramethylthiocycloheptyne sulfoximine was conjugated to SpCas9 on four different residues with varying efficiency and retained protein activity. Conjugation to residue 539 was successful and site-selective while retaining SpCas9 and siRNA bioactivity.

## Abstract 2

SpCas9 was modified to expose an azide to enable conjugation of SpCas9 to siRNA, retaining gene editing and silencing activity.

## Graphical abstract

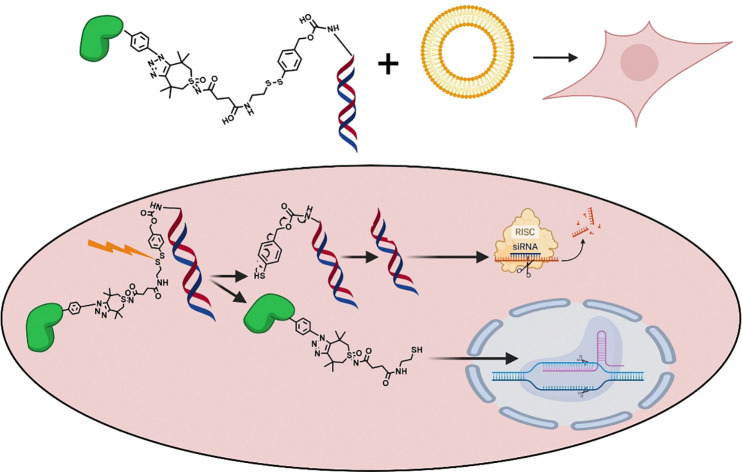

Genome editing using CRISPR–Cas9 and a single guide RNA (sgRNA),^1–3^ or other engineered Cas9 derivatives,^4–8^ has great therapeutic potential for a broad range of genetic diseases. However, broad application of CRISPR–Cas9 is hampered by poor control over gene editing efficiency and outcomes. The most extensively used Cas9 isotype, derived from *S. pyogenes* (SpCas9), is structurally complex, consisting of two endonuclease domains, as well as arginine and lysine rich recognition grooves to bind the sgRNA.^1,9,10^ This complexity limits the engineering options. Controlling the gene editing mechanism mostly involves protein fusions or use of drug compounds which need to be co-delivered into the same cell as SpCas9 to improve the conditions for genome editing *in situ*.^11^ Several protein fusions have successfully achieved high gene editing control^5–8,12,13^ but are limited to a single peptide or protein fragment, as the N-terminus of SpCas9 does not tolerate most protein fusions.^12^ Co-delivery of additional therapeutic molecules is therefore an interesting direction for refinement of gene editing therapies. Current drug delivery vehicles for SpCas9 protein such as lipid, peptide or cell-derived vectors would be difficult to engineer for this purpose as adding additional molecules to these systems would make them more polydisperse and difficult to control.^14–17^ The optimal conditions for encapsulation of RNP and siRNA in LNP are furthermore incompatible. For siRNA the pH needs to be lowered to ensure efficient encapsulation of RNA cargoes^18^ while for RNP encapsulation such pH can lead to protein inactivation.^19^ Surface conjugation can solve this issue by ensuring the two active components are associated together. This can be achieved on lysine or cysteine residues. However, conjugation to one of the 150 lysine residues is likely to lead to protein inactivation due to its abundance in the active domains of SpCas9. The cysteine at position 574 is accessible and has been used successfully in the past as well for conjugation of peptides.^20^ However, the presence of a second cysteine at position 80 in the RuvC-like nuclease domain poses a risk for non-specific conjugation and potential protein denaturation. We aimed to introduce the non-canonical amino acid *p*-azido-*l*-phenylalanine (AzF) in the structure of SpCas9 to generate an SpCas9-drug conjugation platform for azide–alkyne click conjugation.^21–23^

As a proof of concept, we chose to conjugate a small interfering RNA (siRNA) molecule functionalized with tetramethylthiocycloheptyne sulfoximine (TMTHSI), a ring strained alkyne with fast reaction kinetics and high hydrophilicity.^24–26^ A reducible linker molecule utilizing a disulfide moiety enables release of functional siRNA from the protein intracellularly to silence mRNA, while the Cas9 protein would retain its gene editing functionality.^27^ By doing so we would deliver siRNA in the same cell as SpCas9. The proposed conjugation method and subsequent reduction-induced release of siRNA are given in Scheme 1. Conceptually this strategy enables a higher control over the gene editing reaction when siRNA is chosen against, for example, proteins involved in unwanted gene repair mechanisms. In this study we use an siRNA against firefly luciferase (siLuc) as proof of concept. Full methodological details are given in SI Section S1.

**Scheme 1:**
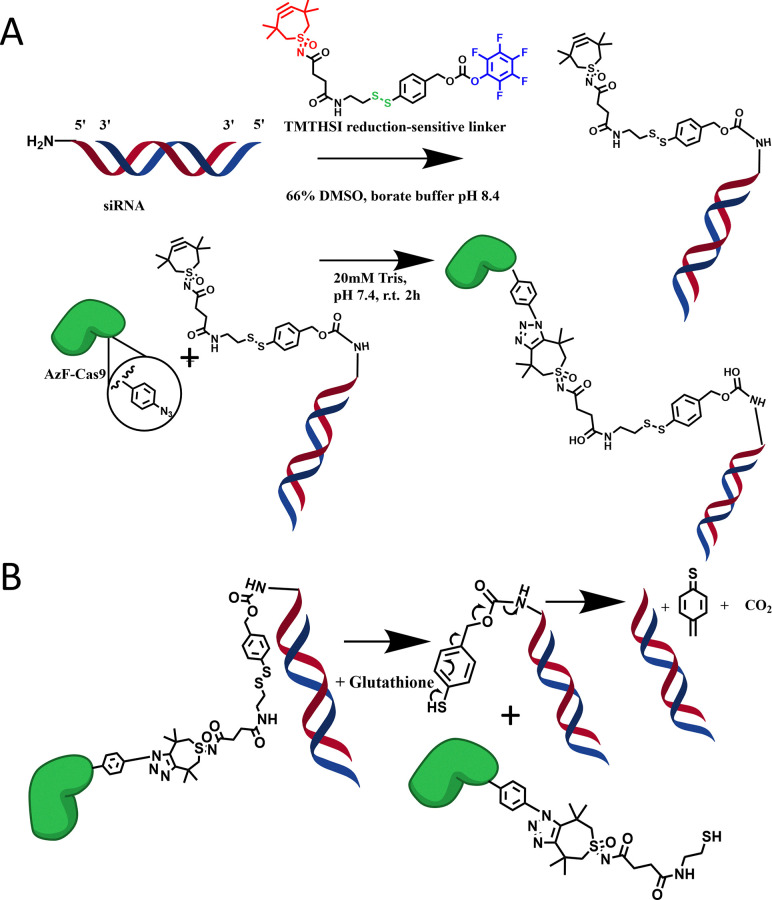
(A) Mechanisms for functionalization of siRNA-amine (sense strand: red, antisense strand: blue) with TMTHSI linked to a reduction sensitive linker, and subsequent “Click” reaction to SpCas9-AzF to yield SpCas9-linker-siRNA conjugates. (B) Release mechanism by reduction of the Cas9-Linker-siRNA conjugate in glutathione leading to native release of the siRNA and a leaving group on the protein surface, constituting a molar weight increase of 358.52 Da compared to unreacted AzF.

The first aim was to express an azide on the surface of SpCas9 without losing protein activity. This azide was introduced as non-natural amino acid during recombinant protein production by Amber STOP codon suppression.^28–30^ SpCas9 was co-expressed with the machinery needed to reprogram the Amber STOP codon to encode AzF in BL21 *E. coli*. This codon was mutated in the SpCas9 production plasmid and the resulting protein was produced, purified by His-tag purification and characterized for size and enzymatic activity. Potential AzF substitution sites were determined *in silico* on SpCas9 crystal structures in the native state (PDB: 4cmp) or complexed with sgRNA and target DNA (PDB: 4un3). First, residues permissive to engineering were chosen which have proven SpCas9 activity after protein domain insertion.^12^ Subsequently, as AzF is aromatic, we chose the aromatic amino-acids (phenylalanine; F, tryptophan; W, tyrosine; Y) were chosen to be substituted in these domains to avoid potential protein misfolding. These engineering-permissive hotspots contained eleven feasible options for amino acid substitution (Fig. S1A). The relative solvent accessible surface area per residue in the native state of SpCas9 was calculated using PyMol (version 2.5) to predict the availability of these amino acids for conjugation. As our aim was conjugation of polyanionic siRNA, we calculated the distance to the closest nucleotide in the complexed state to predict possible steric and electrostatic hindrance siRNA conjugation might have on sgRNA-to-Cas9 complexation and sgRNA-to-target DNA binding. This was calculated using the get_distance function in PyMol between the closest nucleic acid atom (manually checked) in the structure and the relevant residue. These calculations (plotted in Fig. S1A) showed a diverse range of potential residues to substitute with AzF. Four residues were selected for substitution: F196 (highest distance to DNA/RNA), F539 (highest solvent accessibility), Y1036 (moderate solvent accessibility and distance) and F682 (low solvent accessibility, close distance to nucleic acids, close proximity to the “good” predictor F539). F704, which had no solvent accessibility, was explicitly not selected as it is completely hidden in the Cas9 structure. The selected residues were mapped to the SpCas9 structure both in the native and ternary complex states as shown in Fig. S1B and S1C, and represent three distinct surfaces on the structure. Successful substitution of these codons by the TAG amber codon was confirmed by Sanger sequencing (Fig. S2). These proteins were produced successfully by supplementing AzF to the culture medium (Fig. S3) and purified by affinity chromatography and dialysis. The resulting proteins were characterized by SDS-PAGE (Fig. 1A), in which the azide was labelled by DBCO-Alexa Fluor 647 (AF647) at a 10 : 1 molar dye : protein ratio to further show that all four variants contained an azide moiety for conjugation (Fig. 1B). Native SpCas9 did not exhibit AF647 fluorescence after conjugation (Fig. S4). Furthermore, all four variants showed *in vitro* bioactivity, as shown by digesting linearized plasmid DNA (Fig. 1C), indicating that replacing these amino acids with AzF did not inactivate SpCas9 activity. Finally, no prevalent impurities were seen in any of these conditions (Fig. S4).

**Fig. 1:**
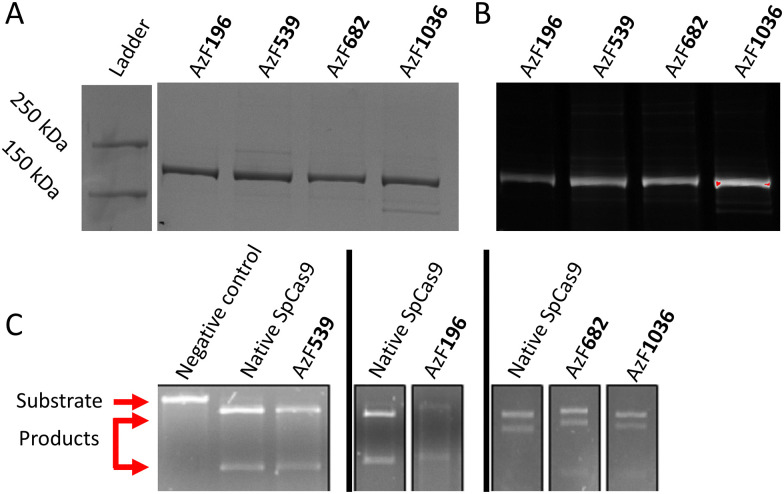
Initial characterization of the four SpCas9-AzF substitutions. (A) SDS-PAGE followed by Coomassie staining to show isolation of the four SpCas9-AzF variants. (B) AF647-DBCO labelled fluorescence picture of the same gel as A. (C) Activity of the four AzF-SpCas9 variants directly after purification, measured by linear pDNA digestion separated by agarose gel electrophoresis. Molecular weights of the undigested pDNA (11 kB; substrate) and digested products due to Cas9 activity (8 kB and 3 kB) are indicated. Each experiment included native SpCas9 as positive control and undigested linear pDNA as negative control.

siLuc-linker-TMTHSI was prepared as reported previously.^24,25^ Functionalized siLuc-linker-TMTHSI was characterized by UPLC, showing that the sense siRNA strand was successfully functionalized^24^ (Fig. S5). Subsequently, siLuc-Linker-TMTHSI was added to AzF-Cas9 at a 2 : 1 molar ratio and incubated at room temperature for 2 hours in tris-buffered saline (300 mM NaCl, 20 mM Tris, pH 7.4), after which any free siLuc-Linker-TMTHSI was removed by dialysis at 50 kDa overnight. This procedure yielded four conjugates with varying conjugation efficiencies, as shown in Fig. 2A. Band intensities were analysed using densitometry analysis in ImageJ to determine the degree of conjugation.^31^ The native protein band and conjugate bands were quantified for each condition, and the relative band intensity of the conjugate band was determined as shown in Fig. S6. This semi-quantitative method was not used as absolute quantitative siRNA conjugation method as baseline peak separation was impossible, but rather as a filter to select a conjugate for further study. SpCas9-196AzF showed a lower conjugation efficiency (35%) compared to the other three conjugates (∼50%). Protein activity was similarly quantified from linear DNA digestions separated on agarose gels. SpCas9-1036AzF showed no discernible nuclease activity (0%) at a similar conjugation efficiency to SpCas9-539AzF and SpCas9-682AzF (60% and 40% retained nuclease activity, respectively) as shown in Fig. 1B. Taken together, SpCas9-539AzF showed the highest conjugation efficiency and nuclease activity, which led to the selection of this variant for further study.

**Fig. 2:**
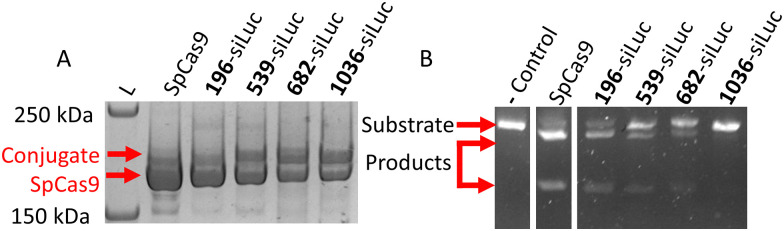
Initial characterization of the siRNA-SpCas9 conjugates on four selected amino acid positions. (A) SDS-PAGE separation and Coomassie stain of all SpCas9 variants incubated with siRNA-linker-TMTHSI for 2 hours. Native SpCas9 (∼160 kDa) and conjugate (∼175 kDa) are indicated. (B) Activity of the conjugates shown in A, measured by linear pDNA digestion of an 11 kB substrate to 8 kB and 3 kB products separated by agarose gel electrophoresis.

After incubation with 5 mM glutathione, which mimics the intracellular reductive environment^32^ to cleave off the linker, complete recovery of the native protein molecular weight is found (Fig. S7). This indicates that the conjugation is reversible in biological conditions, allowing therapeutic co-delivery and subsequent activity of both molecules. This release reaction was further characterized by LC-MS. As reduction of the linker releases native siRNA, a small leaving group is expected on the protein surface (Scheme 1C). The site selectivity of the siRNA conjugation reaction and the presence of the residual group on SpCas9 were further studied by LC-MS after trypsin digestion and glutathione treatment. The peptides containing F539 (KPAFLSGEQK) or the two native Cas9 cysteines were analysed further (see SI.1). First, the substitution of F539 (Fig. 3A) to AzF539 (Fig. 3B) was confirmed by an absolute mass increase of 41.0014 Da corresponding to the substitution of a hydrogen moiety to azide in the peptide normally containing F539. Subsequently, the remaining fragment of the linker and 1,2,3-triazole after release were shown in this peptide by the absolute mass increase of 358 Da to 1502.7374 Da (Fig. 3C). No mass increase was observed for the fragments containing the cysteines, indicating no unspecific thiol–yne conjugate products were formed, further confirming the site-specificity of the siRNA conjugation.^33^

**Fig. 3:**
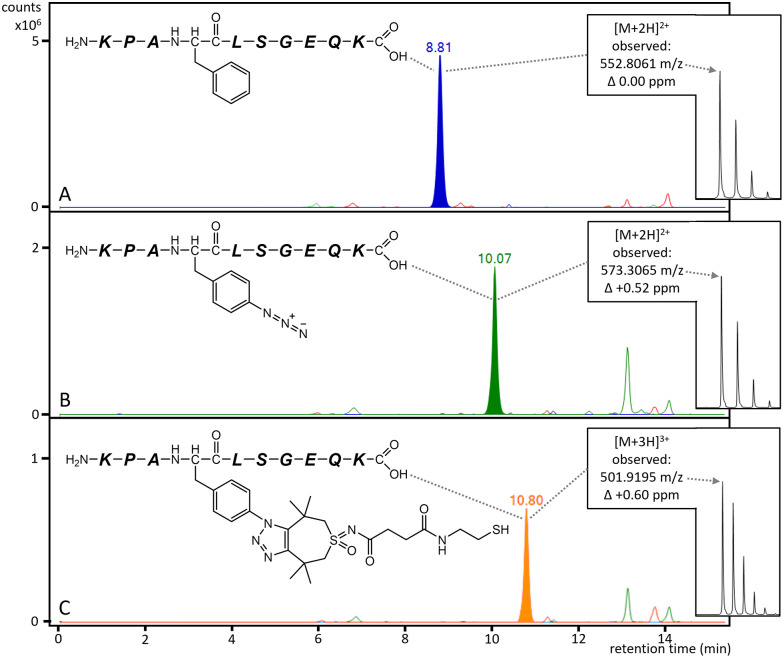
Overlaid LC-MS extracted-ion chromatograms of peptides containing amino acid position 539 after trypsinization. Observed *m*/*z* values and *Δ*_ppm_ relative to theoretical are annotated in each panel. (A) Native Cas9 protein, in which a peptide is found at *m*/*z* 552.8061 [M + 2H]^2+^). (B) AzF539-Cas9, which shows a peptide at *m*/*z* 573.3065 [M + 2H]^2+^), indicating azide substitution. (C) Conjugate treated with 5 mM GSH, which shows a peptide at *m*/*z* 501.9195 [M + 3H]^3+^), corresponding to 539AzF conjugated to TMTHSI and the chemically reduced leftover of the linker.

Finally, the bioactivity of both SpCas9 and siLuc was assessed in two HEK293T reporter cell lines specialized for either molecule. The conjugate was transfected using cationic lipid transfection kits to enable intracellular uptake. This was compared to native SpCas9 or siLuc normalized to the same concentration as the conjugate based on 50% conjugation efficiency measured on gel. The activity of the siLuc was tested in dual Renilla (rLuc) and Firefly luciferase (fLuc) expressing HEK293T cells as shown in Fig. 4A.^34^ Activity is only feasible after intracellular release. The fLuc signal was first normalized to that of rLuc to correct for potential differences in cytotoxicity or metabolic activity between conditions. This normalized fLuc signal was significantly lower for the conjugate and free siRNA than for the negative controls. The Cas9 genome editing efficiency was determined by assessing eGFP knock-out, shown in Fig. 4B.^35,36^ Both the conjugate and native SpCas9 demonstrated eGFP knock-out. Taken together these data indicate that both components are still active after conjugation and co-delivery. Furthermore a pilot experiment was performed in which we co-transfected siRNA targeting different DNA repair targets and native SpCas9 and assessed the specificity of homology directed repair (Fig. S8). This identified that siRNA targeting key DNA repair protein ligase 4, significantly improved HDR specificity 1.3 fold, as expected from previous studies.^37^

**Fig. 4:**
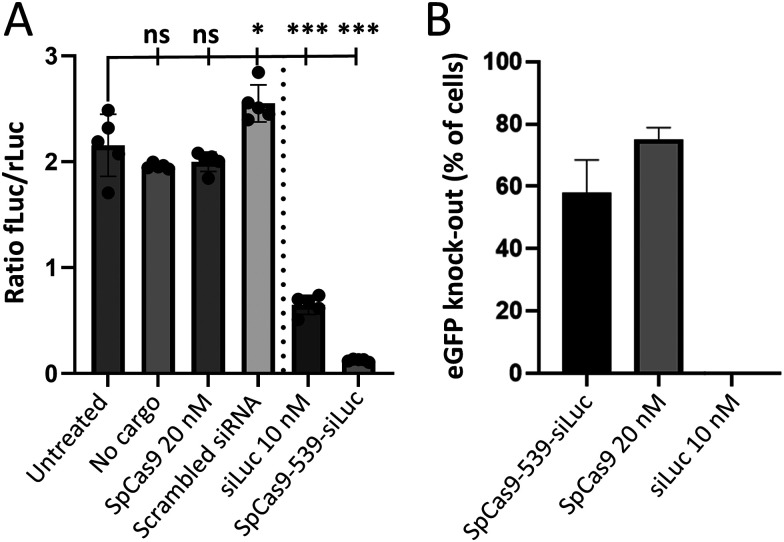
Functional delivery of both siRNA and Cas9 components of SpCas9-539-Linker-siRNA (A) Ratio of fLuc/rLuc signal of untreated cells, controls and SpCas9-siRNA conjugate. *N* = 5 technical replicates (B) Gene editing efficiency for the conjugate compared to the controls. *N* = 3 technical replicates. Ns: not statistically significant (*p* > 0.05). *: *p* < 0.05. **: *p* < 0.01. ***: *p* < 0.001.

We thus conclude that we succeeded at designing an SpCas9-drug conjugation platform based on azide–alkyne click reaction. Substitution of the investigated four aromatic amino acids (F196, F539, F682, and Y1036) on SpCas9 did not inactivate the protein by itself, in line with expectations.^12^ The previously reported TMTHSI and reduction-sensitive linker were applied as a proof of concept for conjugation and subsequent intracellular delivery.^24–26^ siRNA was specifically chosen as model compound as it requires intact delivery in the cytosol to become active. The four conjugation sites showed varied conjugation efficiency and retained protein activity (Fig. 2). The variant with the highest activity and conjugation, Cas9-539-siRNA, was further characterized using LC-MS to show site selectivity of the reaction and the reduction of the siRNA at 5 mM glutathione, consistent with intracellular concentrations (Fig. 3). Furthermore, the Cas9-539-siRNA conjugate showed retained bioactivity for both luciferase silencing (siRNA) and eGFP knockout (SpCas9) activity in cellular activity assays. Other chemical Cas9 engineering studies focused on the direct modulation of Cas9 activity by the conjugated molecule^38–41^ or conjugation to drug carriers.^42–45^ Our platform specifically allows conjugation and subsequent release of any primary amine containing molecule through the TMTHSI-linker chemistry. The release mechanism especially allows us to co-deliver molecules for greater gene editing control such as small molecule drug compounds, peptide or protein fragments, or siRNA targeting relevant intracellular pathways to modulate gene editing in the gene repair, mitosis or histone regulation pathways.^11,37^ It would also enable conjugation to drug delivery vehicles and subsequent release of SpCas9. This proof of concept study demonstrates a versatile click-chemistry based conjugation-and-release platform on the SpCas9 surface and subsequent release while retaining Cas9 functionality. This novel tool can potentially address many outstanding issues in the field of gene editing such as gene editor control and delivery.

## Author contributions

DW: conceptualization, methodology, investigation, formal analysis, writing – original draft, review, editing. MT: conceptualization, methodology, writing – original draft, review. AK: investigation, formal analysis, writing – original draft. ERH: conceptualization, methodology. IRS: investigation, methodology, formal analysis. RMB: methodology, investigation. EB: investigation. OGJ: writing – original draft, review, editing, supervision. TV: resources, writing – review, editing, supervision. EM: conceptualization, resources, writing – review & editing, supervision.

## Conflicts of interest

The authors have no conflicts of interest to declare.

## Supplementary Material

CC-062-D6CC01443G-s001

## Data Availability

Supplementary information (SI): 1: detailed materials and methods. Fig. S1: conjugation site selection process. Fig. S2: gene and amino acid alignment of SpCas9 and the mutants used in this work. Fig. S3: titration of AzF supplementation for SpCas9-AzF production. Fig. S4: full length SDS-PAGE gels corresponding to Fig. 2A and B. Fig. S5: UPLC chromatograms detailing siRNA functionalization. Fig. S6: SDS-PAGE gel densitometry workflow for conjugation efficiency quantification. Fig. S7: SDS-PAGE gel demonstrating removal of siRNA after GSH treatment. Fig. S8: screening siRNA molecules for knockdown of NHEJ mediators. See DOI: https://doi.org/10.1039/d6cc01443g.
