# Supplementary material for: Azide-functionalized SpCas9 enables generation of site-selective and bioactive Cas9-siRNA conjugates
Source: Chem Commun (Camb). 2026 Jun 2;62(48):12084–8. doi: 10.1039/d6cc01443g (PMC13232376; doi:10.1039/d6cc01443g)
Supplement: CC-062-D6CC01443G-s001 [file CC-062-D6CC01443G-s001.pdf]

Supplementary Information (SI) for Chemical Communications.
This journal is © The Royal Society of Chemistry 2026

Supplementary information for "Azide-functionalized SpCas9 enables generation of site-selective and bioactive Cas9-siRNA conjugates"

Supplementary information 1:

Detailed materials and methods

General reagents

All chemicals were obtained from Sigma Aldrich/Merck (Zwijndrecht, The Netherlands) unless specified otherwise. All PCR primers were obtained from Integrated DNA Technologies (Leuven, Belgium), and their sequences are noted in Supplementary Table 1. SpCas9 was produced using pSP-Cas9 (Addgene #62731) as previously published, with modifications to the protocol noted where applicable (1). sgRNA and ssDNA templates were acquired from Merck (Haverhill, United Kingdom) and applied as published previously (1). Sequences are given in Supplementary Table 1. TMTHSI was kindly provided by Cristal Therapeutics (Maastricht, The Netherlands).

Table S1: DNA primers, sgRNA, template DNA and siRNA sequences used throughout this work.

Site directed mutagenesis primers	
**F196, Forward**	gcattgatcgggttttcttcctacagctgattataggtttgc
**F196, Reverse**	gcaaacctataatcagctgtaggaagaaaacccgatcaatgc
**F539, Forward**	tttctgttcgccactcagctaagccggtttgcgcataccc
**F539, Reverse**	gggtatgcgcaaaccggcttagctgagtggcgaacagaaa
**F682, Forward**	gcaaagccgtccgatttcagctaatctagaatcgttttgcctgattg
**F682, Reverse**	caatcaggcaaaacgattctagattagctgaaatcggacggctttgc
**Y1036, Forward**	catgatgtttgaatagaaaaactatttggcggtcgctttgcc
**Y1036, Reverse**	ggcaaagcgaccgccaaatagtttttctattcaaacatcatg
Sequencing primers	
**F196**	gcccagtagtaggttgaggc
**F539 and F682**	tgacgatctggacaacctgc
**Y1036**	cgaaaacacccagctgcaaa
CRISPR materials	
**sgRNA targeting sequence**	gcugaagcacugcacgccgu
**ssODN HDR template**	caagctgcccgtgccctggcccaccctcgtgaccaccctgagccacggcgtgcagtgcttcag ccgctaccccgaccacatgaagc
siRNA*	
**siFL, Sense**	5'- (NH2C6)GfgAfuGfaAfgUfgGfaGfaUfuAfgUf(invdT) -3'
**siFL, Antisense**	5'- dAsCfuAfaUfcUfcCfaCfuUfcAfuCfcdTsdTs(C6NH)(Rho3B-NHS) -3'
**siLuc, Sense**	5'-(NH_2C_6)sascCfgaaAfGfGfucUfuaccGfgas(invdT)-3'
**siLuc, Antisense**	5'-UfsCfscgguaagaccuUfucggususu-3'

*siRNA legend:
Af, Gf, Uf, Cf: 2'-Fluoro nucleotide
a, g , u, c: 2'-O-methyl-nucleotide
S: phosphorothioate

(invdT): inverted dT
Rho3B-NHS: Rhodamine 3B fluorescent label (ex 566/em 589) introduced via NHS coupling prior to hybridization.

In silico SpCas9 structural analysis

Pymol (version 2.5) was subsequently used to determine the solvent-accessibility of the aromatic amino-acids in the structure of native SpCas9 (Protein Databank (PDB) accession number 4cmp), in the hotspots outlined in the first design constraint. This was used as predictor for conjugation efficiency, as it describes the 3D accessibility of the amino acid in question. In addition, the distance between the amino acids and the DNA or RNA nucleotides in the ternary complex of SpCas9 (PDB file 4ump) was measured using the PDB structure viewer (accessible from: https://www.rcsb.org/3d-view, accessed February 2023). Four amino acids were selected for substitution based on a different pattern in these two parameters as outlined in the Results section: F196, F539, F682 and Y1036. Visualizations of these amino acids highlighted on the SpCas9 apoprotein and ternary complexes were made using the PDB structure viewer, where the protein was visualized at 50% size in the cartoon representation and the DNA, RNA and selected amino acids were visualized as molecular surfaces to emphasize the distance between the DNA/RNA and modified amino acids in the structures.

STOP codon reprogramming and SpCas9-AzF recombinant expression

AzF was introduced in SpCas9 through amber STOP-codon reprogramming using an artificial tRNA synthetase/tRNA pair derived from *M.jannaschii*, encoded on pEVOL_pAzF (Addgene #31186) (2). This plasmid was transformed into BL21 *E.coli* (New England Biolabs, Ipswich, USA) using heat shock transformation at, followed by chloramphenicol selection using 25 µg/mL. After selection, these BL21-pEVOL_pAzF cells were made chemically competent through calcium chloride treatment and stored at -80 °C until further use.

pSP-Cas9 was used as plasmid for recombinant SpCas9 expression and as such modified for SpCas9-AzF expression. It was mutated to harbor the TAG stop codon to substitute one of the candidate amino acids using the Agilent Site Directed Mutagenesis XL 2 kit (Agilent Technologies, Amstelveen, The Netherlands) according to the manufacturer's specifications and using the mutagenesis primers noted in the Supplementary Table 1. Mutagenized plasmids were transformed into XL10 Gold Ultracompetent *E.coli* (Agilent Technologies) for amplification. Subsequently, mutagenized pDNA was recovered from individual clones using the GenElute Plasmid Miniprep kit (Thermo Fischer Scientific, Eindhoven, The Netherlands). Sanger sequencing was performed by Macrogen (Amsterdam, The Netherlands), using primers noted in Supplementary Table 1. Alignments were prepared and visualized using Benchling.com (accessed February 2023). Appropriately mutated plasmids were transformed into the BL21-pEVOL_pAzF cells by heat shock at 42 °C for 30s. After recovery, bacteria positive for pEVOL_pAzF and the mutagenized pSP-Cas9 were selected using both chloramphenicol and ampicillin resistance.

SpCas9-AzF expression and purification were done as published previously, except that expression was performed using BL21 *E.coli* (New England Biolabs) in Luria Broth supplemented with 500 µM IPTG and 2 mM AzF (Santa Cruz Biotechnology, Heidelberg, Germany) (1). Subsequent to production,

the AzF-SpCas9 was dialyzed against 300 mM NaCl, 15 mM Tris, 0.1 mM EDTA, pH 7.4 and supplemented with 10% glycerol. Samples were stored at -80 °C until further use.

SpCas9-AzF in vitro activity assays

In vitro functionality of SpCas9-AzF variants, prior and after conjugation, was determined by DNA digestion as published previously (1). Briefly the protein was complexed to sgRNA targeting eGFP and incubated with linearized pDNA (pMJ922, Addgene #78312) for two hours in a reaction mixture containing NEBuffer 3.1 (New England Biolabs), Ribolock RNAse inhibitor (Thermo Fischer Scientific) and nuclease free water (Integrated DNA Technologies). Following this, the protein complex was degraded using 20 U/mL proteinase K for 30 minutes at ambient temperature. Digestion products were separated by agarose gel electrophoresis for 1 hour on a 1% TAE agarose gel at 75 volts and visualized using UV transillumination on a ChemiDoc™ XRS+ imager (Bio-Rad Laboratories, Veenendaal, The Netherlands). Protein activity was quantified using gel densitometry analysis measured in ImageJ (version 1.49p), normalized to unmodified recombinantly produced SpCas9 protein.

SDS-PAGE

SpCas9-AzF purity was assessed qualitatively using SDS-PAGE. Samples were incubated with 1x Laemmli buffer (Bio-Rad Laboratories), containing 55 mM dithiothreitol at 70°C for 10 minutes. Bolt 4 to 12% gradient Bis-Tris 1.0 mm gels (Thermo Scientific, Landsmeer, the Netherlands) were used to separate the proteins at 150V for 55 minutes. PageRuler Plus Prestained Protein Ladder (Thermo Scientific) was used to determine the molecular weight of the bands alongside an unreacted SpCas9 as control. The gels were then stained using PageBlue Protein Staining (Thermo Scientific, Landsmeer, the Netherlands) and subsequently imaged using a ChemiDoc™ XRS+ imager using the Coomassie Blue preset (far red epifluorescence, 715/30 emission filter).

Alexa Fluor 647-DBCO (AF647-DBCO; Thermo Fischer) was incubated with SpCas9-AzF at a 10:1 molar ratio of dye:protein at ambient temperature overnight to confirm the azide functionality. Subsequently, the reaction mixtures were separated using SDS-PAGE as noted above, and imaged using the ChemiDoc™ XRS+ imager using the Alexa Fluor 647 preset (red epifluorescence, 700/50 emission filter).

siRNA functionalization, conjugation and characterization

Two siRNA sequences were used in this study. The first was siRNA targeting an arbitrary gene (*AHA1*), labelled with rhodamine 3B on the 3' side of the antisense (guide) strand of the siRNA (Axolabs, Kulmbach, Germany) for convenient fluorescent monitoring (siFL). The second siRNA sequence targeted firefly luciferase (siLuc) (Axolabs, Kulmbach, Germany). Both sequences contained a primary amine for functionalization on the 5'end of the sense (passenger) strand of the siRNA. Details on the sequences and chemical modifications of the siRNA are given in Supplementary Table 1.

Both siRNA's with an amine handle on the 5'end of the sense strand were functionalized with a reduction-sensitive click linker (3) as previously described (4). In brief, approximately 3 equivalents of linker (0,68 µmol) dissolved in 1 mL of DMSO was added to one equivalent of siRNA (0,23 µmol) dissolved in 0.5 mL of 100 mM borate buffer pH 8.4 and stirred for 15 minutes. UHPLC using a hexylamine-based method described in Supplementary Table 2 was used to confirm reaction completion, and the reaction mixture was subsequently purified via spin filters with a 5 kDa MWCO and a PD10 desalting column.

Supplementary Table 2: UPLC method details for TMTHSI-Linker-siRNA characterization.

UPLC system:	Waters H-Class
UPLC column:	Acquity BEH C18 1.7 µm, 100 x 2.1 mm
Eluent A:	100mM Hexylamine acetic acid
Eluent B:	90% ACN 10% 100mM Hexylamine acetic acid
Needle flush solvent:	ACN:MQ 90:10
Sample tray Temperature:	20° C
Column Temperature:	89° C
Flow:	0.3 mL/min
Injection volume:	5.0 µL
UV Detection:	258 nm
Detection mode	2D
Sampling rate	10 points/sec
Filter time constant	Normal
Resolution	1.2 nm
Exposure time	Auto
Run time:	10 minutes

Time (min)	Flow (mL/min)	% A	% B
0	0.3	60	40
0.50	0.3	60	40
5.00	0.3	45	15
5.50	0.3	15	85
7.00	0.3	15	85
7.50	0.3	60	40
10.00	0.3	60	40

siRNA-L15-TMTHSI was added to AzF-Cas9 at a 2:1 molar ratio and incubated at room temperature for 2 hours in tris-buffered saline (300 mM NaCl, 20 mM Tris, pH 7.4). The conjugate was dialyzed using 100-200 µL Micro Float-a-Lyzer casettes with a 50 kDa MWCO (Repligen, Breda, The Netherlands) against tris-buffered saline with added glycerol (300mM NaCl, 20mM Tris, 10% glycerol, pH 7.4 and stored at -20°C) to remove unreacted siRNA-linker-TMTHSI until further use.

AzF-Cas9 conjugated to siRNA was assessed by SDS-PAGE separation and analysis. Conjugates were denatured in Laemmli buffer without DTT, and separated on a Novex™ WedgeWell™ 6%, Tris-Glycine gel (Thermo Scientific, Landsmeer, the Netherlands) for one hour at 150V. Samples containing Rhodamine 3b-labelled siRNA were analysed using the Rhodamine setting of the ChemiDoc^{TM} XRS+ imager (green epifluorescence, 602/50 emission filter). Following this, the gel was incubated with PageBlue staining overnight and washed with ddH2Oto de-stain the non-protein components in the gel. The gel was imaged using the Coomassie Blue setting of the ChemiDoc^{TM} XRS+ imager (far red epifluorescence, 715/30 emission filter). Conjugation efficiencies were approximated using gel densitometry analysis in ImageJ (version 1.49p) by quantifying the surface area of the main SpCas9 band (160 kDa) and the larger protein species in the gel (corresponding to conjugates). The conjugation efficiency was defined to be the surface area of the bands above 160 kDa as percentage of the sum of their surface areas. This was rounded to the nearest percentage divisible by 5 (e.g. 5, 10, 15, etc) to account for the semi-quantitative accuracy of the quantification technique (5).

To assess the release of the siRNA from the SpCas9, samples were incubated with 5 mM L-glutathione (GSH) for 30 minutes at 37 °C prior to SDS-PAGE to mimic intracellular reducing conditions (6).

Trypsin digest and LC-MS characterization of the conjugation site and release kinetics

SpCas9, SpCas9-539AzF, and SpCas9-539-siRNA (0,05 mg each) were dissolved in 50 mM ammonium bicarbonate (pH 7.8). Trypsin (from bovine pancreas, >10.000 BAEE U/mg protein) was then added at a 1:20 (w/w) ratio and samples were incubated overnight at 37 °C with light agitation. Post-digestion, samples were reduced by adding 5 mM GSH for 1 hour at 37 °C.

For purification and desalting, C18 solid phase extraction cartridges (Avantor™ 7020-02 BAKERBOND™ spe Octadecyl) were used. The cartridges were preconditioned with 1 mL acetonitrile and 1 mL 0.1% trifluoroacetic acid (TFA) in water. Tryptic digests were acidified to pH <4 using 5% TFA, centrifuged at 10.000 x *g* for 2 minutes, and loaded onto the cartridges. Cartridges were washed with 0.7 mL 0.1% TFA in water, and peptides eluted using 0.7 mL 80/20 acetonitrile/water with 0.1% TFA. The eluates were dried under nitrogen gas and reconstituted in 5% acetonitrile in water for LC-MS, with a 10 µL injection volume.

The LC-MS setup comprised an Agilent 1260 Infinity LC and a 6560 IM-QTOF mass spectrometer (Agilent Technologies, Santa Clara, USA). Separation was achieved on a Waters ACQUITY UPLC HSS T3 column (100 mm x 2.1 mm, 1.8 µm particles) with a matching VanGuard pre-column (5 mm x 2.1 mm, 1.8 µm particles). The column temperature was maintained at 40 °C, and the mobile phase consisted of water with 0.1% formic acid (A) and acetonitrile with 0.1% formic acid (B) at a flow rate of 0.2 mL/min. The elution program involved a 3-minute isocratic hold at 5% B, followed by a 25-minute linear gradient to 60% B and a subsequent 5-minute linear increase to 95% B.

The eluate was ionized using an electrospray source in positive mode at 3500 V capillary and 2000 V nozzle voltages. Nitrogen served as both nebulizing (40 psi, 11 L/min) and drying gas (11 L/min), set at temperatures of 350 °C and 300 °C, respectively. The IM-QTOF mass range was set between 100–1700 m/z. Data was internally calibrated to reference masses m/z 121.0509 and 922.0098 and processed using Agilent IM-MS Data File Reprocessing Utility (Version 10.00) and PNNL PreProcessor software (version 4.0). Feature identification was executed by the 'find features' (IMFE) option of the Agilent IM-MS Browser (version 10.0) using the 'Unbiased' isotope model, a charge state limit of 5, and a minimum ion intensity of 100. Filtering was performed based on m/z (300–1700) and a minimum abundance of 500, defined as the 'max ion volume' (arbitrary units, a.u.).

Generated feature lists were matched to the m/z values of anticipated ions from the theoretical tryptic peptide 'KPAFLSGEQK' in its native form, azido-Phe, and reduced conjugate forms, with a permitted error margin of 5 ppm. The dominant ionic species used in the analysis were $[M+nH]^{n+}$, with sodium adducts present in trace amounts.

Following the characterization of anticipated ions, the presence other expected but unwanted side products were assessed. Based on literature, thiol-yne addition of alkynes and cysteines could result in a stable thiol enol ether moiety even under reducing conditions (7). To scout for this, peptides ICYLQEIFSNEMAK (position 79-92, [M] 1687.7949 Da) and IECFDSVEISGVEDR (position 572-586, [M] 1696.7614 Da) were assessed due to their free cysteine residues. Similarly to the abovementioned data processing strategy, LC-MS feature lists were generated to search for peptide without missed cleavages and 1 missed cleavage at the N- or C-terminus of the peptides.

Generating HEK293T-fLuc-rLuc cell line

In order to read out siRNA activity, a HEK293T reporter cell line was produced expressing firefly luciferase (fLuc) and renilla luciferase (rLuc). pLenti PGK V5-LUC Neo (w623-2) was used for expression of fLuc and pLenti.PGK.blast-Renilla_Luciferase was used for expression of rLuc respectively. Both were acquired from Addgene (Addgene # 21471 and # 74444 respectively). Lentiviral particles were prepared as published previously using pRSV-Rev (Addgene #12253), pMD2.G (Addgene #12259) and pMDLg-pRRE(Addgene #12251) for lentivirus production as described previously (1).

HEK293T cells were cultured in low glucose DMEM medium and transduced first using rLuc lentivirus. Cells were selected using 5 ug/mL blasticidin for 7 days prior to transduction using fLuc lentivirus to achieve co-expression. Cells were selected using 1mg/mL G418 (Thermo Fischer Scientific) and 5 µg/mL blasticidin (Invitrogen). Cells were cultured for 1 month after transduction in presence of selection antibiotics to ensure gene expression. rLuc and fLuc expression were assessed using the Promega Dual Glo luciferase assay kit (Promega, Leiden, The Netherlands) according to the manufacturer's specifications until the respective signals remained stable over time.

Gene editing and silencing assays in reporter cells

HEK293T-eGFP cells were used to measure SpCas9 gene editing as reported previously (1,8). Briefly, cells were seeded in 96 well plates 24 hours prior to genome editing at a density of 10,000 cells/well. SpCas9 and conjugates were transfected into the cells using the ProDeliverIN CRISPR kit (OZ biosciences, Marseille, France). SpCas9, sgRNA and HDR template DNA were used as published previously in a 1:1:2 molar ratio prior to transfection, and ProDeliverIN CRISPR was added in a 1 µg protein to 3.3 µL of transfection reagent (1). Other steps in the protocol were performed as specified by the kit manufacturer. siRNA was added to cells as negative control with the same concentration and sequence as the conjugated siRNA. . Genome editing induced by the NHEJ and HDR pathways was measured after 5 days by flow cytometry on the BD FACS Canto II (Becton Dickinson, New Jersey, USA) in the FITC and Pacific Blue channels, respectively.

siRNA functionality was tested on silencing of firefly luciferase in the fLuc+rLuc HEK293T cells (siRNA sequences in Supplementary Table 1). The Lipofectamine RNAiMAX kit (Thermo Fischer Scientific) was used for transfection of the siRNA or the SpCas9-siRNA conjugate. The Promega Dual Glo luciferase assay System was used for measuring the firefly and Renilla luciferase activity.

Briefly 10.000 fLuc+rLuc HEK293T cells were seeded in 96 wells Cellstar white flat bottom well plates standard TC surface (Greiner Bio-One, catalogue number 655083) in 90 µl medium (DMEM low glucose + 10 FCS). 20 hours after seeding cells were transfected according the manual of the thusing 10 µl mixture containing 0.3 µl Lipofectamine RNAiMAX and 1 pmol the siRNA or the SpCas9-siRNA in Opit-MEM medium . 48 hours after transfection) luminescence was measured using the Promega Dual Glo luciferase assay System on a Mithras LB940 plate reader (Berthold Technologies, Vilvoorde, Belgium) using 1 seconds of counting time. The signal of firefly luciferase was normalized to the signal of renilla luciferase to account for differences in cell protein expression and proliferation between conditions, expressed as a ratio between fLuc/rLuc. Controls were scrambled non-targeting siRNA (MISSION® siRNA Universal Negative Control #1, Sigma Aldrich), SpCas9 protein (negative for siRNA) or positive control siRNA.

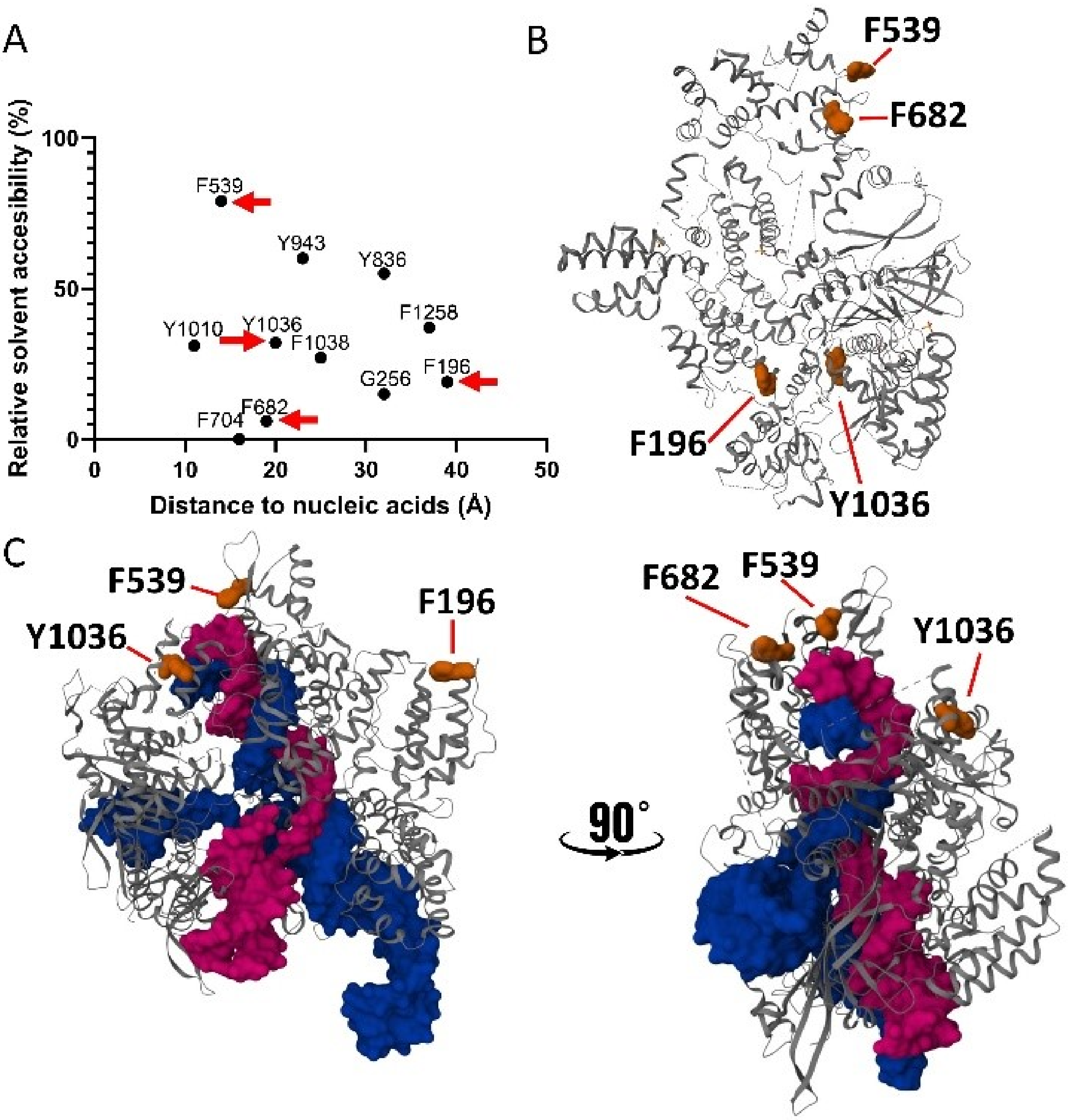

Supplementary data figures

Figure S1: Rationale behind the amino acid sites engineered in the SpCas9 protein in this work. A: The atomic distance of aromatic amino acids in SpCas9 to the sgRNA or DNA in the complex, calculated from PDB 4ump, plotted against their sasa calculated from PDB 4cmp. B: Selected amino acids to be substituted with AzF mapped to the 3D structure of native SpCas9 (gray, pdb 4cmp. C: The same residues mapped to the SpCas9 ternary complex. Gray: SpCas9, Blue: target DNA, Pink: sgRNA.

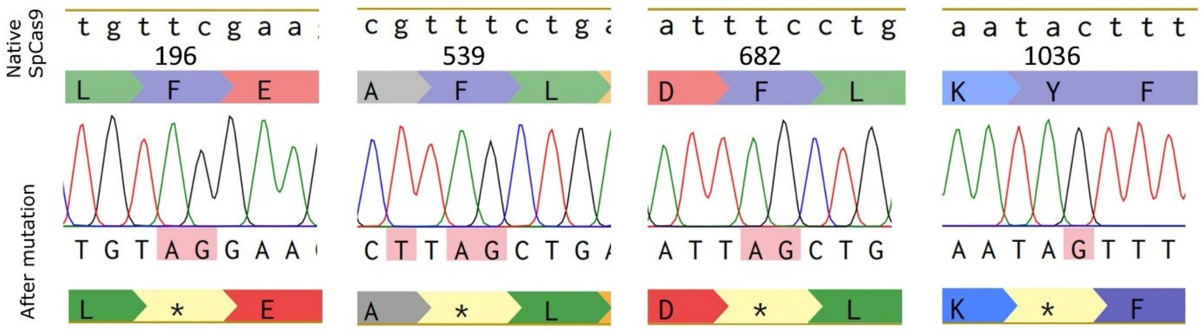

Figure S2: Gene sequencing and amino acid alignments for each STOP codon (*) programmed in the PL-SpCas9 production plasmid to introduce AzF. The substituted amino acid number is indicated.

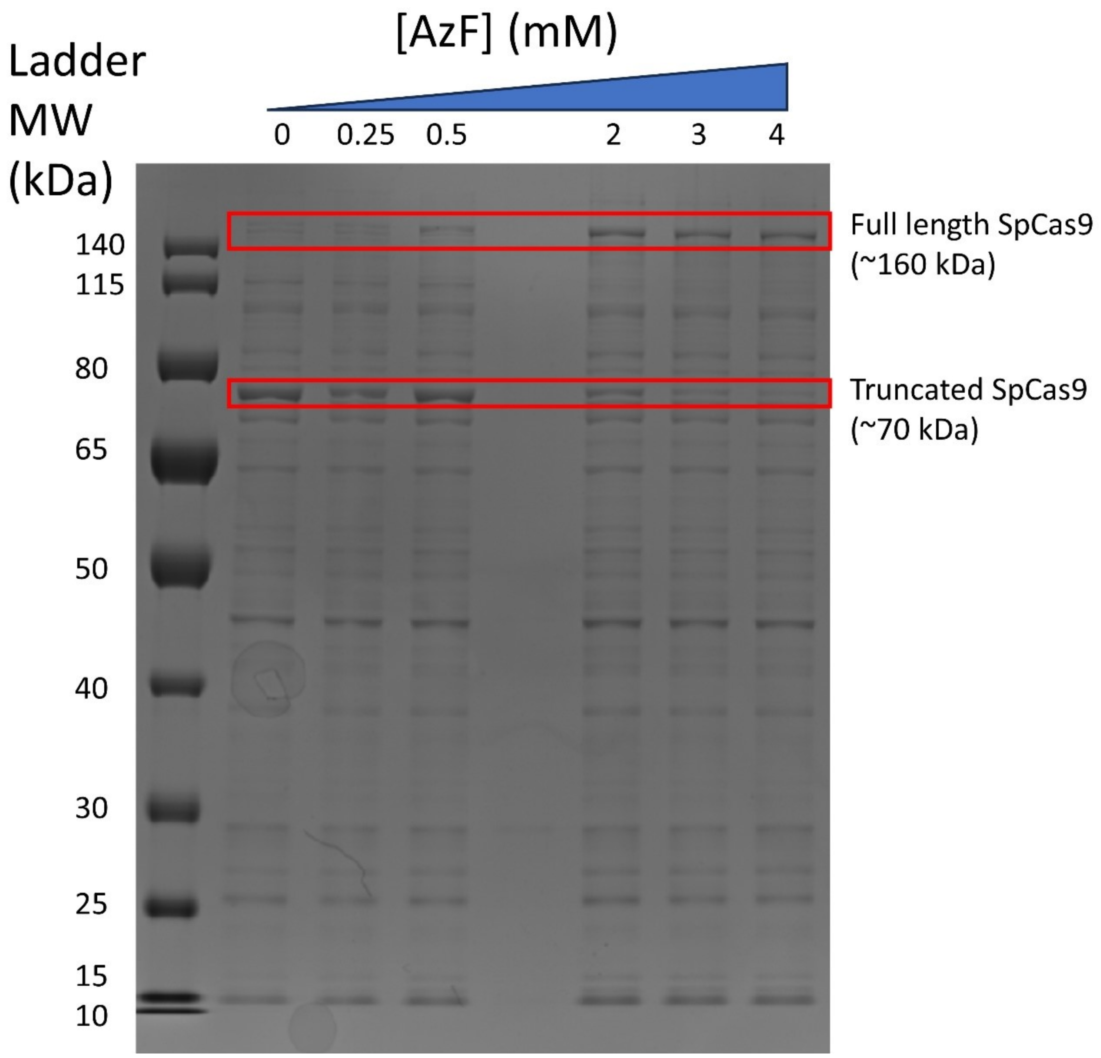

Figure S3: Optimization of AzF concentration in culture medium of *E. coli* for stop codon suppression of 682AzF-SpCas9, to be used for production of AzF-SpCas9 . Bacterial lysates were separated on an SDS-PAGE gel and stained using for SpCas9 purity determination. pSpCas9 with a STOP codon at position 682 was used in this optimization.

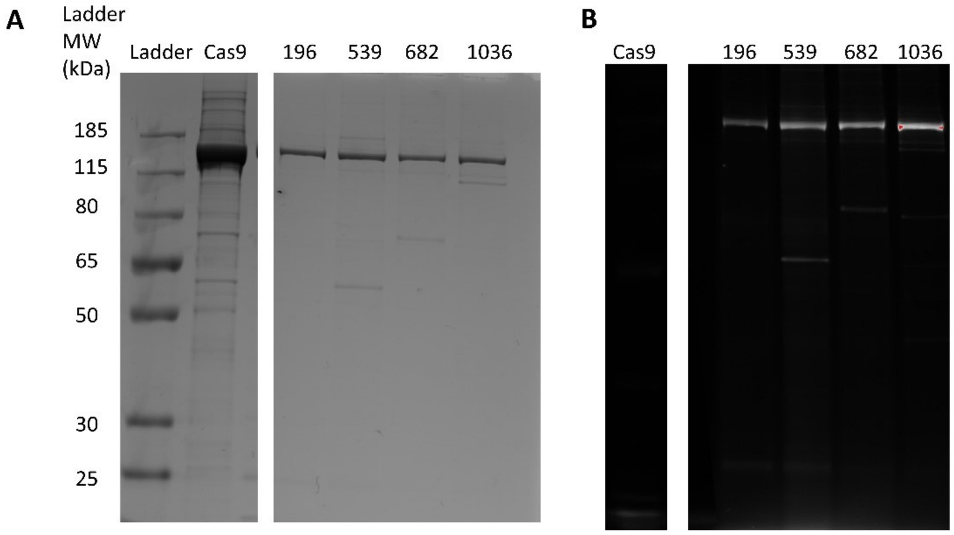

Figure S4: Full-length SDS-PAGE gels. No appreciable protein bands are seen above 160 kDa, indicating that the AzF-SpCas9 after defrosting prior to use is stable and not exhibiting photo-crosslinking, as is possible with the use of this amino acid. Slight impurities are visible, but negligible compared to the full length protein band. **B:** DBCO-AF647 conjugated to all four variants and native Cas9 and visualized on SDS-PAGE (same gel as A, prior to Coomassie staining).

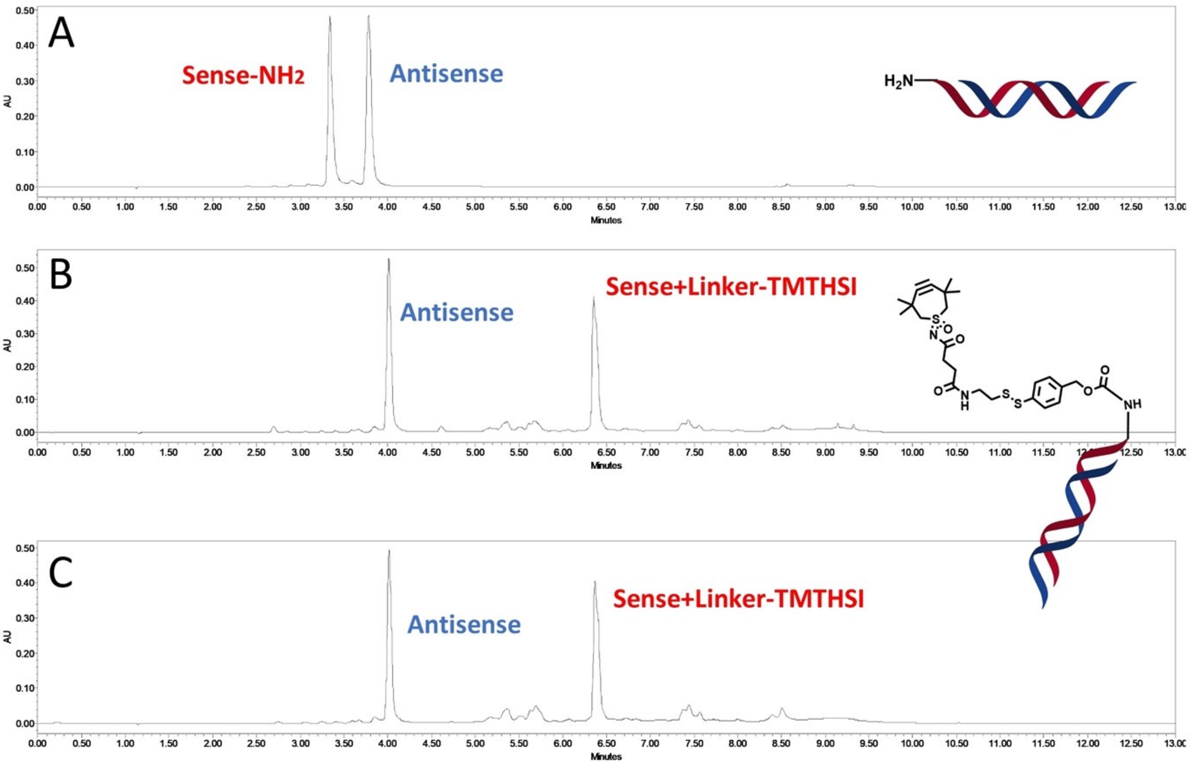

Figure S5: UPLC chromatograms for functionalized siRNA. The sense (modified) and antisense strand peaks are indicated. A: Free siRNA-NH2 duplex before functionalization. B: Functionalized siRNA before purification. C: Functionalized siRNA after purification.

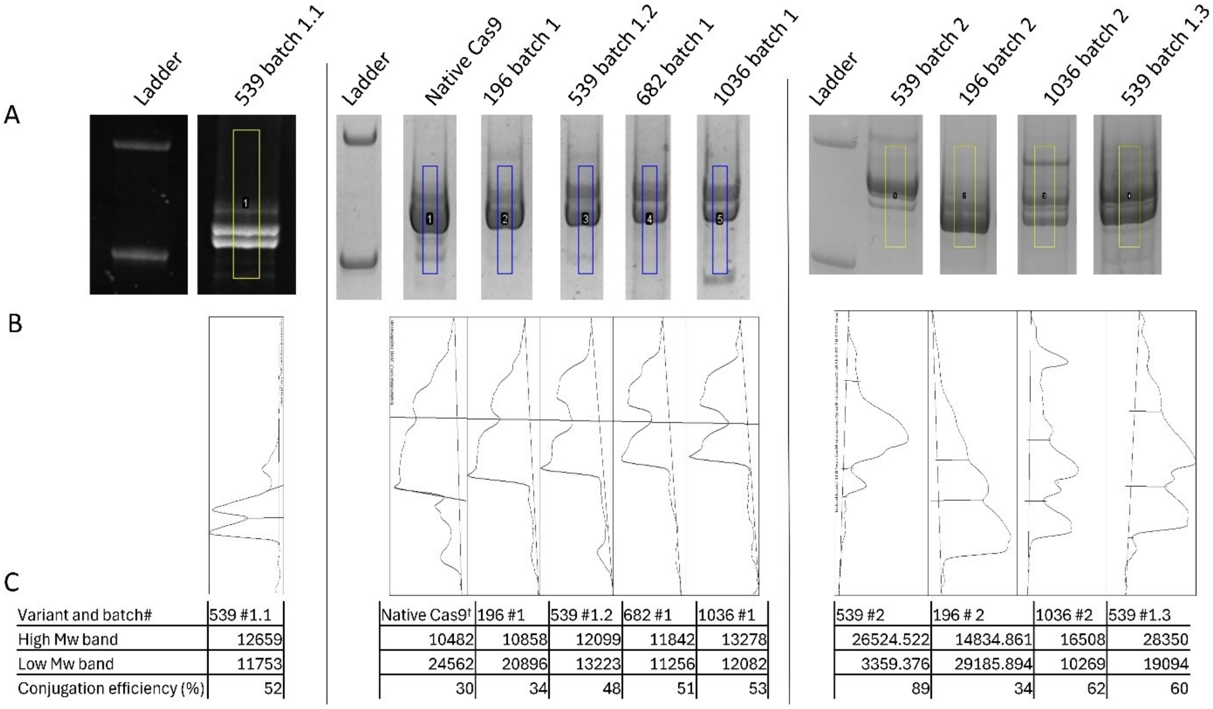

C

Variant and batch#	539 #1.1	Native Cas9†	196 #1	539 #1.2	682 #1	1036 #1	539 #2	196 # 2	1036 #2	539 #1.3
High Mw band	12659	10482	10858	12099	11842	13278	26524.522	14834.861	16508	28350
Low Mw band	11753	24562	20896	13223	11256	12082	3359.376	29185.894	10269	19094
Conjugation efficiency (%)	52	30	34	48	51	53	89	34	62	60

Figure S6: Quantification of conjugation efficiency using gel densitometry. ImageJ was used to plot signal density, and the resulting peaks were integrated using the built in wand tool. Conjugation efficiencies were calculated by the following formula: Area_HighMw / (Area HighMw+AreaLowMw)*100%. A: replicate measurement gels of different batches of conjugated siRNA conjugates. ImageJ signal densities are determined per relevant lane. C: Peak integration corresponding to the gel lanes in B. C: Calculated conjugation efficiencies. For variant 539 the same batch was measured in triplicate, leading to a mean ± SD of 53.3% ± 6.1%. Rounded down to account for the high variation, we took this as an efficiency of 50% in downstream calculations.

†: Protein band was overloaded leading to a likely overestimate of the real conjugation efficiency. *: Protein band was smeared leading to a likely overestimation of the real conjugation efficiency.

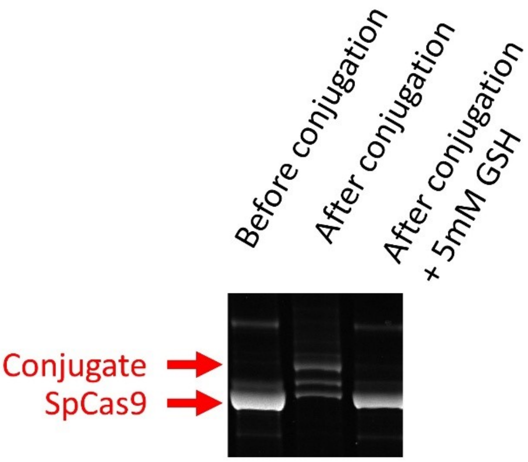

Figure S7: SDS-PAGE gel showing unconjugated 539AzF-SpCas9, conjugate 539siRNA-SpCas9 and 539siRNA-SpCas9 treated with GSH for 30 minutes.

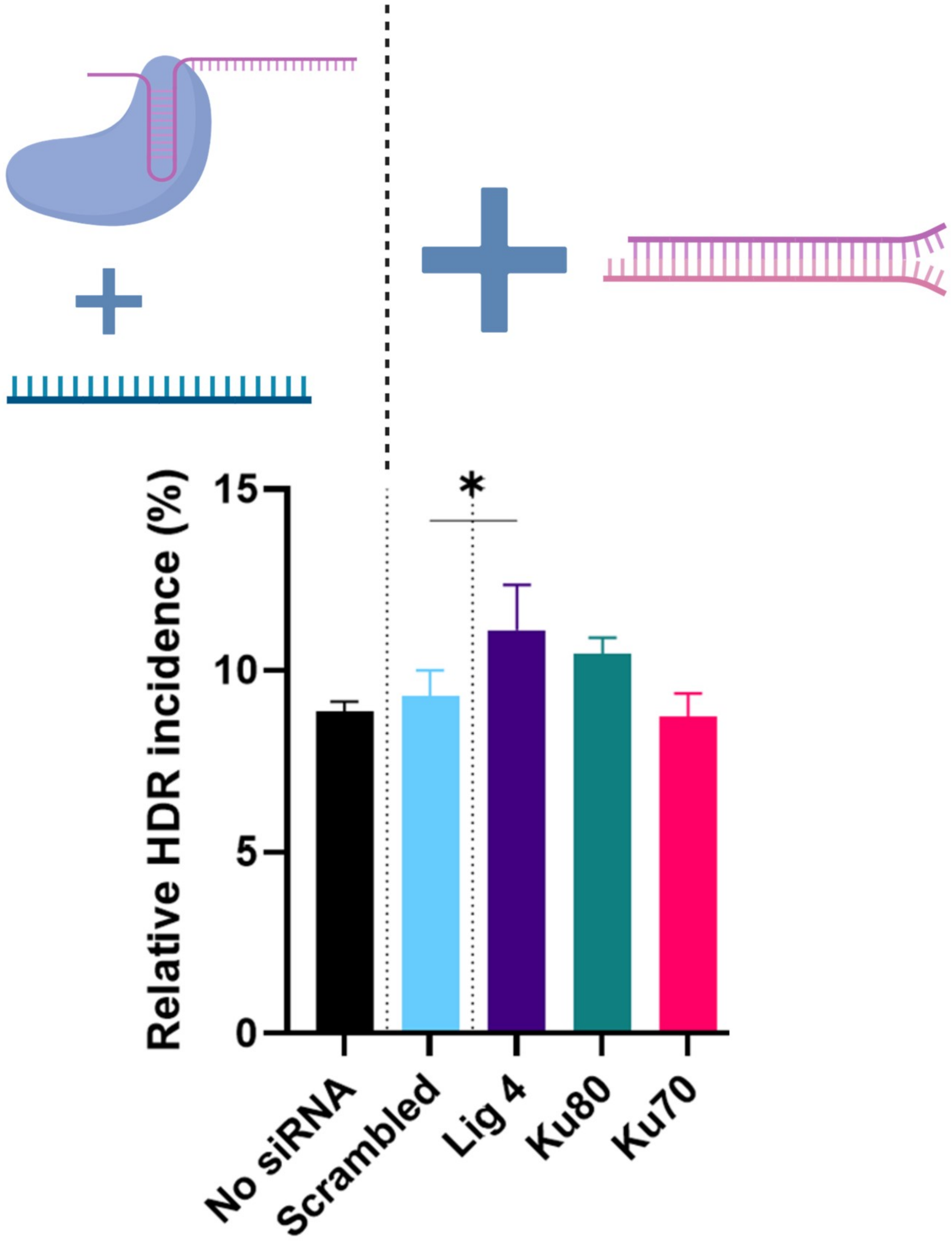

Figure S8: HDR gene editing assay (1) using SpCas9+HDR template DNA performed in absence and presence of additional siRNA molecules targeting major proteins in the non-homologous end-joining pathway. If NHEJ is inhibited sufficiently, the relative HDR incidence increases. A one-way ANOVA was done with post hoc comparisons between the Scrambled control and the three siRNA test cases. *: p<0.05.

References used in Supplementary Information

1. Walther J, Wilbie D, Tissingh VS, Öktem M, van der Veen H, Lou B, et al. Impact of Formulation Conditions on Lipid Nanoparticle Characteristics and Functional Delivery of CRISPR RNP for Gene Knock-Out and Correction. Pharmaceutics. 2022;14(1):213.
2. Chin JW, Santoro SW, Martin AB, King DS, Wang L, Schultz PG. Addition of p-Azido-l-phenylalanine to the Genetic Code of Escherichia coli. J Am Chem Soc. 2002 Aug 1;124(31):9026–7.
3. Hebels ER, Dietl S, Timmers M, Hak J, van den Dikkenberg A, Rijcken CJF, et al. Versatile Click Linker Enabling Native Peptide Release from Nanocarriers upon Redox Trigger. Bioconjug Chem. 2023 Dec 11;
4. Timmers M. Innovative cleavable and bioorthogonal conjugation chemistries for application in core-crosslinked polymeric micelles and bioconjugates [PhD thesis]. [Utrecht]: Utrecht University; 2023.
5. Alonso Villela SM, Kraïem H, Bouhaouala-Zahar B, Bideaux C, Aceves Lara CA, Fillaudeau L. A protocol for recombinant protein quantification by densitometry. Microbiologyopen. 2020 Jun;9(6):1175–82.
6. Deponte M. Glutathione catalysis and the reaction mechanisms of glutathione-dependent enzymes. Biochim Biophys Acta. 2013 May;1830(5):3217–66.
7. Zhang C, Dai P, Vinogradakeov AA, Gates ZP, Pentelute BL. Site-Selective Cysteine–Cyclooctyne Conjugation. Angewandte Chemie International Edition. 2018 May 28;57(22):6459–63.
8. Glaser A, McColl B, Vadolas J. GFP to BFP Conversion: A Versatile Assay for the Quantification of CRISPR/Cas9-mediated Genome Editing. Mol Ther Nucleic Acids. 2016 Jul 12;5(7):e334.
